# Efficacy of Narrative Nursing Model in psychological adjustment and improvement of treatment compliance in hypertension patients

**DOI:** 10.12669/pjms.41.12.12894

**Published:** 2025-12

**Authors:** Litao Du, Yiming Shao, Yubin Chi, Yingying Wei, Hongna Han

**Affiliations:** 1 Litao Du, Department of Cardiovascular Diseases 2, Hebei Provincial Hospital of Chinese Medicine, Shijiazhuang, Hebei Province 050000, P.R. China; 2 Yiming Shao, Department of Cardiovascular Diseases 2, Hebei Provincial Hospital of Chinese Medicine, Shijiazhuang, Hebei Province 050000, P.R. China; 3 Yubin Chi, Department of Cardiovascular Diseases 2, Hebei Provincial Hospital of Chinese Medicine, Shijiazhuang, Hebei Province 050000, P.R. China; 4 Yingying Wei, Department of Cardiovascular Diseases 2, Hebei Provincial Hospital of Chinese Medicine, Shijiazhuang, Hebei Province 050000, P.R. China; 5 Hongna Han, Department of Cardiovascular Diseases 2, Hebei Provincial Hospital of Chinese Medicine, Shijiazhuang, Hebei Province 050000, P.R. China

**Keywords:** Hypertension, Narrative nursing model, Psychological adjustment, Treatment compliance

## Abstract

**Objective:**

To explore the efficacy of the narrative nursing model on psychological adjustment and improvement of treatment compliance in patients with hypertension.

**Methodology:**

A retrospective analysis was conducted on the clinical data of 120 patients with hypertension who were admitted to Hebei Provincial Hospital of Chinese Medicine between January 2022 to January 2025. The nursing interventions and pre-/post-assessments had been implemented as part of a quality improvement initiative in the hospital, and this study retrospectively reviewed the medical records and outcome data of patients who received different nursing approaches. Among them, 56 patients received routine nursing (control group) and 64 patients received the narrative nursing model on the basis of routine nursing (observation group). Psychological adjustment, treatment compliance, blood pressure levels and nursing satisfaction were compared between the two groups.

**Results:**

After intervention, the Self-Rating Depression Scale (SDS) and Self-Rating Anxiety Scale (SAS) scores of both groups decreased compared to pre-intervention and were significantly lower in the observation group than the control group (P<0.05). The scores for medication compliance, exercise training, dietary regulation, stress management and blood pressure monitoring in both groups increased after the intervention, with markedly higher scores in the observation group compared to the control group (P < 0.05). Post-intervention diastolic and systolic blood pressure decreased in both groups. Patients in the observation group reported considerably lower post-intervention blood pressure levels (P<0.05). Nursing satisfaction in the observation group (96.88%) was higher than that in the control group (85.71%) (P<0.05).

**Conclusion:**

Implementing the narrative nursing model for patients with hypertension can improve psychological status, alleviate depression and anxiety, enhance treatment compliance, optimize blood pressure control and achieve high patient satisfaction.

## INTRODUCTION

The World Health Organization estimates that approximately 1.2 billion adults worldwide suffer from hypertension, with a trend toward younger ages.^1^,^2^ Hypertension is a major risk factor for cardiovascular and cerebrovascular diseases. It contributes to complications such as stroke and coronary artery disease, reduces patients’ quality of life, and adds considerable socioeconomic burden.^3^,^4^ Since effective control of hypertension relies on patients’ adherence to a long-term, regular medication regimen, a healthy lifestyle and self-monitoring of blood pressure, thus, improving treatment compliance is critical for effective hypertension control.^5^,^6^

Studies have found that psychological factors play a significant role in the occurrence, development and treatment of hypertension. Negative emotions such as anxiety and depression can affect blood pressure regulation through the neuroendocrine system, leading to blood pressure fluctuations and increasing treatment difficulty.^7^ Additionally, a poor psychological state is often associated with lowered treatment compliance.^8^ However, traditional nursing models primarily focus on disease care and health education, often neglecting patients’ psychological needs, which may limit their impact on psychological adjustment and treatment compliance.^9^,^10^ As an emerging nursing model, narrative nursing focuses on patient-centered care, based on listening to, understanding and responding to patients’ experiences.^11^

This approach enables patients to explore their inner feelings and needs, helping them reconstruct positive disease cognition and coping strategies.^12^ Existing studies suggest that narrative nursing can enhance psychological well-being and treatment adherence in patients with chronic or oncological conditions.^13^,^14^ However, the value of narrative nursing in psychological adjustment and treatment compliance improvement for patients with hypertension is still unclear. Recent international research has further demonstrated the benefits of narrative-based interventions across various healthcare contexts. White and Epston’s classic framework of narrative therapy highlights “externalization” and “re-authoring” as key strategies for helping patients reconstruct illness meaning and strengthen their sense of agency.^15^ Sha and Pi reported that narrative nursing significantly improved treatment adherence and self-management among elderly patients with diabetes.^16^ Similarly, Xue et al. showed that narrative medicine training enhanced empathy, professionalism, and humanistic care among nursing students.^17^ These findings provide both theoretical and empirical support for the broader application of narrative nursing in chronic disease management, including hypertension.

This study aimed to explore the efficacy of the narrative nursing model in patients with hypertension, providing a scientific basis for optimizing hypertension nursing programs and improving patients’ health management levels.

## METHODOLOGY

Clinical data from 120 patients with hypertension admitted to Hebei Provincial Hospital of Chinese Medicine between January 2022 to January 2025 were retrospectively selected. Among them, 56 patients received routine nursing (control group) and 64 patients received narrative nursing on the basis of routine nursing (observation group).

### Ethical approval:

The study was approved by the Ethics Committee of Hebei Provincial Hospital of Chinese Medicine on September 30, 2021 (No. HBZY2021-KY-070-01).

### Inclusion criteria:

- Patients meet the diagnostic criteria for hypertension.^13^
- Aged >18 years.
- Clear consciousness, able to communicate normally, with certain language expression ability.
- No severe cognitive impairment or mental illness.
- Able to cooperate with psychological assessments and qustionnaires.
- Resident locally for convenient management.
- Complete clinical data.

### Exclusion criteria:

- Patients with severe failure of vital organs (heart, liver, kidney, etc.).
- Patients with severe chronic diseases such as malignant tumors or hematological diseases.
- Presence of language, hearing, or visual impairments affecting narrative nursing implementation.
- Extremely poor compliance, unable to complete the study.
- Participating in other clinical trials that may affect the study results.
- Pregnant or lactating women.

All enrolled patients completed the full course of nursing interventions and outcome assessments. No participants dropped out during the study period. This complete retention was attributed to the inpatient setting, where the entire intervention was delivered before discharge, and to the strict exclusion criteria that removed patients with poor compliance, severe cognitive impairment, or communication barriers. Therefore, an Intention-to-Treat (ITT) analysis was not applicable in this study.

### Nursing approach:

### Routine nursing included:

### Health education:

Knowledge about the etiology, pathogenesis, hazards, prevention and treatment of hypertension was provided to patients and their families through the distribution of publicity brochures and health lectures. This intervention aimed to enhance patients’ disease understanding and promote self-management.

### Dietary nursing:

To control salt, protein and fat intake. The patients were informed that their daily salt intake should not exceed 5-gram from all sources, including soy sauce, pickled vegetables and ham. The consumption of high-salt foods was restricted and the use of the quantitative salt spoon was recommended. Patients were instructed to restrict the intake of animal oil, fatty meat, fried foods, choose edible oils rich in unsaturated fatty acids such as olive oil and camellia oil and control the daily intake at 25 - 30 g. Patients were advised to consume at least 300 g of fresh vegetables and 200 g of fruits per day and to supplement their diet with minerals such as potassium and magnesium, which help lower blood pressure. Patients were instructed to select high-quality proteins such as lean meat, fish, beans and bean products to ensure balanced nutrition.

### Exercise guidance:

Patients were instructed to engage in regular aerobic exercises, such as walking, jogging, Taijiquan, or swimming, at least five days a week, for 30-60 minutes per day. The exercise intensity should be such that the heart rate during exercise does not exceed (170-age) beats/min. In case of discomfort, such as dizziness and palpitations during exercise, the exercise should be stopped immediately. Patients with poor physical conditions were advised to reduce the intensity of their exercise and extend the duration of their exercise. Generally, the intensity of the exercise should lead to slightly increased perspiration and faster breathing that does not affect communication.

### Smoking cessation and alcohol restriction:

Patients were encouraged to quit smoking; they were informed of the smoking-related risk to the cardiovascular system, such as blood vessel constriction and increased blood pressure. Nicotine replacement therapies, such as smoking cessation candies and patches, as well as professional smoking cessation institutions, were recommended when necessary. Patients were also encouraged to restrict alcohol consumption to not exceed 25g for men and 15g for women.

### Medication guidance:

Patients received detailed information about the name, dosage, usage, effects and adverse reactions of the antihypertensive drugs used. For example, calcium channel blockers may cause facial flushing and ankle edema; angiotensin-converting enzyme inhibitors may cause dry cough, etc. Patients were instructed to seek medical attention in case of adverse effects and not to stop or change drugs by themselves. The importance of taking medicine on time and in the correct dosage as prescribed by the doctor, avoiding missing doses, randomly increasing or decreasing the dosage or stopping the medicine by themselves, was emphasized. Medicine boxes or mobile phone reminder functions were recommended to help patients take medicine regularly.

### The narrative nursing model:

In the observation group, narrative nursing was delivered in addition to routine care. Each patient received approximately 4 to 6 narrative nursing sessions over a period of two to three weeks, tailored to individual psychological needs and length of stay. Each session lasted 30 to 40 minutes, conducted in a quiet, private setting to facilitate open communication. The intervention was administered by senior registered nurses with at least five years of clinical experience, who had completed certified training in psychological counseling and narrative-based nursing, and who were supervised by a clinical psychologist. A standardized internal protocol was used to ensure consistency, and regular weekly case discussions and peer reviews were held to maintain intervention fidelity and quality control. In addition to the routine nursing regimen, the following aspects were included:

### Externalization:

Separating the disease from the patient using techniques, such as:

### Dialogue guidance:

Using metaphorical language to help patients visualize hypertension. For example, “If hypertension were seen as an external ‘intruder’ in your life, how would you describe it? How has it affected your emotions and daily behavior?”

### Visual expression:

Providing drawing papers or cards and encouraging patients to describe the image of “hypertension” (such as “a red monster”, “a heavy stone”) with pictures, symbols, or keywords and share its characteristics.

### Relationship definition:

Guiding patients to think, “How does this ‘guy’ of hypertension affect your life choices? Is your relationship with its confrontation, compromise, or something else?”, helping patients realize that the disease is an external challenge rather than a label of themselves.

### Deconstruction:

Analyzing the meaning behind the patients’ experience.

### In-depth listening:

Gain an in-depth understanding of patients’ experiences through open-ended questions. For example, “When hypertension appeared for the first time, what other important things happened in your life?” “What are you most worried about losing with the appearance of this ‘guy’?”

### Emotional decoding:

Identifying the potential emotional needs in patients’ narratives. For example, the sense of loneliness behind giving up social activities due to the disease, or the sense of helplessness due to the side effects of medication. Responding with empathetic language: “It sounds like you have made a lot of sacrifices to control your blood pressure. These grievances must have been bottled up in your heart for a long time, right?”

### Sorting out contradictions:

Helping patients discover contradictions and conflicts in their narratives. For example, “You hope to control your blood pressure through exercise, but you are worried that your body can’t handle it. This contradiction makes you very torn, right?”

### Rewriting:

Reconstructing a positive narrative.

### Looking for exception events:

Guiding patients to recall moments of successfully managing blood pressure or breaking through difficulties. For example, “Was there a time when your blood pressure suddenly became stable and you felt like you ‘defeated’ it? What did you do then?”

### Strengthening positive actions:

Magnifying exceptional events and endowing patients with a “hero role”. For example, “After you actively adjusted your diet that time, your blood pressure dropped, which shows that you are completely capable of taking control of your health. This accomplishment is truly praiseworthy!”

### Constructing a new storyline:

Planning future actions based on positive elements. For example, formulating a “one-week small health goal” (walk for 10 minutes every day, reduce one high-salt diet) and discussing the life changes after achieving the goal (such as having more energy to accompany family members).

### External witnesses:

Creating supportive group resonance.

### Peer witness meeting:

Organizing patients to share stories and set up a “witness” role. For example, after one patient tells about the experience of relieving blood pressure fluctuations through meditation, other patients give recognition and feedback with phrases like “I hear the efforts you have made for your health, which makes me also want to try new methods”.

### Family participation:

Inviting family members as “life witnesses” to share the progress of patients in disease management (such as actively measuring blood pressure, persisting in exercise) and strengthening patients’ self-efficacy.

### Medical staff affirmation:

Recording patients’ behavior changes (such as blood pressure reaching the standard for seven consecutive days) by the nursing staff and publicly affirming them during ward rounds: “Your persistence has made the treatment effect remarkable. This self-discipline is worth our learning!”

### Treatment documents:

Solidifying the achievements of narrative nursing.

### Story file production:

Sorting out patients’ narrative content, externalization works (such as paintings), records of exception events and making a “healthy growth manual”, marking key time nodes and the change process.

### Commitment contract:

Formulating a personalized health plan with patients, including short-term goals (such as daily salt intake < 5-grame) and long-term visions (such as participating in community hiking activities) that both parties sign to confirm.

### Result visualization:

Using charts to record data such as blood pressure changes, exercise check-ins and mood scores, combining them with narrative content to form an illustrated “health narrative map”, update it regularly and provide it for patients to review.

The design of the narrative nursing model in this study was grounded in the theoretical principles proposed by White and Epston, who conceptualized narrative therapy as a process of externalization, deconstruction, and re-authoring of personal experiences.^15^ These principles provided the framework for guiding patient dialogue and reconstructing disease cognition.

### Observation Indicators:

The collected indicators included:

- The psychological adjustment status of the two groups, as measured by the Self-Rating Depression Scale (SDS) and the Self-Rating Anxiety Scale (SAS). The SDS score ranged from 53 - 62 points for mild depression, 63 - 72 points for moderate depression and ≥ 73 points for severe depression. The SAS score ranged from 50 - 59 points for mild anxiety; 60 - 69 points for moderate anxiety and ≥ 69 points for severe anxiety.
- Treatment compliance of the two groups, as assessed before and after the intervention using the Self-Management Behavior Assessment Scale for Hypertensive Patients, which evaluated medication adherence, exercise training, diet regulation, stress management and blood pressure monitoring. The score range for each dimension was 0-20 points, with a higher score indicating better treatment compliance.
- Blood pressure levels of the two groups before and after the intervention, including diastolic blood pressure and systolic blood pressure.
- The nursing satisfaction of the two groups, as based on the Newcastle Nursing Satisfaction Scale (NSNS), with a total of 95 points: very satisfied (≥ 76 points), satisfied (61 - 75 points), basically satisfied (41 - 60 points) and dissatisfied (≤ 40 points). Very satisfied, satisfied and basically satisfied were included in the total satisfaction score.

### Statistical Analysis:

Data were analyzed using SPSS 25.0. Measurement data were described by Mean ± SD and analyzed by the t-test. Count data were described by frequency and composition ratio (%) and analyzed by the ***χ^2^*** test. A P-value < 0.05 indicates a statistically significant difference.

## RESULTS

There were no statistically significant differences in general data between the two groups (P > 0.05) Table-I. Furthermore, all patients in both the control and observation groups completed the assigned nursing interventions and outcome assessments, resulting in a 100% completion rate. There was no significant difference in the SDS and SAS scores between the two groups before the intervention (P > 0.05). After the intervention, the SDS and SAS scores of both groups were lower than those before the intervention and the scores of the observation group were lower than those of the control group (P < 0.05), as shown in Table-II.

**Table-I:** Comparison of General Data between the two Groups.

Items	Observation Group (n=64)	Control Group (n=56)	t/χ^2^	P
***Gender [n (%)]***				
Male	33(51.56)	31(55.36)	0.173	0.678
Female	31(48.44)	25(44.64)		
Age (Mean±SD, years)	55.25±7.16	53.81±8.02	1.039	0.301
Course of Disease (Mean±SD, years)	9.11±3.15	8.59±3.71	0.830	0.408
***Education Level [n (%)]***				
Junior High School or Below	22(34.38)	25(44.64)	1.939	0.379
Senior High School	29(45.31)	24(42.86)		
College or Above	13(20.31	7(12.50)
Body Mass Index (Mean±SD, kg/m^2^)	22.69±1.98	23.05±2.07	0.973	0.333
***Hypertension Classification [n (%)]***				
Grade 1	17(26.56)	16(28.57)	0.116	0.944
Grade 2	33(51.56)	29(51.79)		
Grade 3	14(21.88)	11(19.64)		
***Marital Status [n (%)]***				
Married	55(85.94)	49(87.50)	0.063	0.802
Unmarried/Widowed/Divorced	9(14.06)	7(12.50)		
***Underlying Diseases [n (%)]***				
Diabetes	7(10.94)	4(7.14)	0.517	0.472
Coronary Heart Disease	11(17.19)	8(14.29)	0.189	0.664
Others	5(7.81)	3(5.36)	0.289	0.591

**Table-II:** Comparison of Psychological Adjustment Status between the Two Groups (Mean±SD, Scores).

Time	Group	Number of Cases	SDS	SAS
Before Intervention	Observation group	64	59.79±3.71	61.72±4.46
	Control Group	56	60.46±4.06	60.98±5.17
	*t*		0.944	0.842
	*P*		0.347	0.402
After Intervention	Observation group	64	45.71±4.19	46.93±3.94
	Control Group	56	50.06±3.88	51.35±4.28
	*t*		5.872	5.889
	*P*		0.000	0.000

Pre-intervention treatment compliance of both groups was comparable (Table-III). After the intervention, the scores of medication treatment, exercise training, diet regulation, stress management and blood pressure tracking in both groups were higher than those before the intervention and the scores of the observation group were higher than those of the control group (P < 0.05) and as shown in Table-IV, before the intervention, the diastolic and systolic blood pressures were similar in the two groups (P > 0.05). Interventions resulted in a significant decrease in blood pressure levels in both groups, with the levels in the observation group being markedly lower than in the control group (P < 0.05). The narrative nursing approach was associated with a considerably higher nursing satisfaction (96.88%) compared to the routine nursing (85.71%) (P < 0.05) Table-V.

**Table-III:** Comparison of Treatment Compliance between the Two Groups (Mean±SD, Scores).

Time	Group	Number of cases	Medication treatment	Exercise training	Diet regulation	Stress management	Blood pressure tracking
Before Intervention	Observation group	64	13.21±1.08	13.25±1.35	12.41±1.46	14.63±1.42	12.77±1.26
	Control Group	56	12.96±1.27	12.81±1.56	12.27±1.19	14.48±1.57	13.01±1.11
	*t*		1.165	1.656	0.571	0.550	1.100
	*P*		0.246	0.100	0.569	0.584	0.274
After Intervention	Observation group	64	18.68±1.20	18.33±1.26	18.40±1.35	18.18±1.24	18.71±1.05
	Control Group	56	17.28±1.32	17.41±1.01	17.30±1.27	17.24±1.22	16.99±1.36
	*t*		6.085	4.371	4.577	4.174	7.804
	*P*		0.000	0.000	0.000	0.000	0.000

**Table-IV:** Comparison of Blood Pressure Levels between the Two Groups (Mean±SD, mmHg)

Time	Group	Number of Cases	Diastolic Blood Pressure	Systolic Blood Pressure
Before Intervention	Observation group	64	102.97±6.98	150.57±8.79
	Control Group	56	104.15±7.19	151.24±9.06
	*t*		0.911	0.411
	*P*		0.364	0.682
After Intervention	Observation group	64	92.33±5.41	138.86±7.72
	Control Group	56	96.17±6.64	143.13±8.10
	*t*		3.489	2.954
	*P*		0.001	0.004

**Table-V:** Comparison of Nursing Satisfaction between the Two Groups [n (%)]

Group	Number of Cases	Very Satisfied	Satisfied	Basically Satisfied	Dissatisfied	Total Satisfaction
Observation group	64	36(56.25)	17(26.56)	9(14.06)	2(3.13)	62(96.88)
Control Group	56	27(48.21)	14(25.00)	7(12.50)	8(14.29)	48(85.71)
*χ^2^*						4.870
*P*						0.027

## DISCUSSION

This study compared the routine nursing and narrative nursing models, systematically evaluating their impacts on the psychological state, treatment compliance and blood pressure control of patients with hypertension. The results show that the narrative nursing model can improve psychological status, alleviate depression and anxiety, enhance treatment compliance and optimize blood pressure control in patients with hypertension. This nursing approach is also associated with markedly higher patient satisfaction.

Poor long-term blood pressure control can lead to damage to vital organs, such as the heart, brain and kidneys, significantly affecting patients’ quality of life.^18^ Exploring effective nursing intervention models to improve the psychological adjustment ability and treatment compliance of hypertension patients is, thus, crucial for optimizing the effect of blood pressure control. The results of this study show that narrative nursing was more efficient than the routine approach in lowering the SDS and SAS scores of patients with hypertension, confirming that this mode of nursing can effectively improve their psychological adjustment ability.

Hypertension patients often experience anxiety and depression due to the long-term and complex nature of the disease.^19^ Narrative nursing uses “externalization” techniques to help patients re-examine the disease from an objective perspective. At the same time, the “deconstruction” process enables a deep exploration of the underlying needs behind patients’ emotions, allowing suppressed emotions to be released. “Rewriting” guides patients to focus on their own positive experiences and reshapes their confidence in managing the disease.^20^ This process aligns with the core concept of “re-authoring” in narrative therapy theory, which enables patients to redefine their identity in relation to the illness and regain a sense of control and agency.^15^ In narrative nursing research on patients with chronic diseases, Sha et al. and Pi et al.^16^ found that engaging patients in illness storytelling and reflective dialogue effectively enhanced self-management and reduced psychological distress. These international perspectives are consistent with our results and further confirm the adaptability of narrative-based nursing interventions. As Greenhalgh emphasized, storytelling should be applied where it can deliver the greatest added value—particularly in chronic diseases, where patients’ identity and lived experience strongly influence their engagement and compliance.^21^,^22^ In contrast to the narrative nursing approach, traditional routine nursing focuses more on the technical aspects of disease management. It pays insufficient attention to patients’ psychological needs, resulting in poor psychological adjustment effects, which further highlights the unique value of narrative nursing in psychological intervention.^23^

These psychological improvements may be explained through established theoretical frameworks. According to Bandura’s Self-Efficacy Theory, verbal persuasion, mastery experiences, and emotional reassurance—core elements of narrative nursing—can enhance a patient’s belief in their ability to manage illness.^24^ In parallel, White and Epston’s Narrative Therapy model supports the idea that “externalization” helps patients detach their identity from the disease, while “re-authoring” promotes emotional release and cognitive restructuring.^15^ Furthermore, narrative nursing promotes an empathetic and dialogical approach that aligns with Bordin’s Therapeutic Alliance Theory, which posits that trust, shared goals, and mutual understanding between providers and patients are key to engagement and treatment adherence.^25^ Collectively, these models provide a theoretical foundation for the observed improvements in psychological adjustment and treatment compliance.

In terms of treatment compliance, the scores of the observation group in dimensions such as medication treatment, exercise training and diet regulation were significantly higher than those of the control group (P < 0.05), confirming that narrative nursing helps improve the treatment compliance of patients with hypertension. Through the “external witness” link, narrative nursing organizes patients to share their disease management experiences, forms a peer support network and enhances patients’ self-efficacy. The establishment of “treatment documents” visualizes patients’ progress and continuously motivates them to actively participate in their treatment.^26^ A study by Sukartini T et al.^23^ on diabetic patients showed that narrative nursing can significantly improve self-management behaviors of patients, further supporting the results of this study. In contrast to the narrative approach, routine nursing mainly employs one-way health education, lacking the stimulation of patients’ internal motivation, which results in a limited effect on improving compliance.

The application of the narrative nursing model in this study effectively improved patients’ compliance with the treatment plan through multi-dimensional interventions, laying a solid foundation for blood pressure control, which is considered a key indicator for evaluating the effectiveness of hypertension nursing. This study demonstrates that, following the intervention, the reduction ranges of diastolic blood pressure and systolic blood pressure in the observation group were more pronounced than those in the control group (P < 0.05), indicating that the narrative nursing intervention model is more effective in blood pressure control in patients with hypertension. It is plausible that narrative nursing reduces blood pressure fluctuations caused by anxiety and stress by improving patients’ psychological state.

At the same time, higher treatment compliance ensures the effective implementation of medication treatment and lifestyle interventions and multiple approaches work together to promote blood pressure to reach the standard. Ali SH et al.^26^ found in their nursing research on patients with hypertension that narrative nursing, combined with behavioral guidance, can significantly reduce blood pressure levels. This study further confirms the advantages of narrative nursing in integrating psychological and behavioral interventions. In addition, the nursing satisfaction of patients who received narrative nursing in this study was as high as 96.88%, significantly higher than that of the control group (85.71%) (P < 0.05). Such an effect may be primarily due to the “listening-empathy-response” communication model in narrative nursing, which significantly enhances patients’ sense of identity and trust in nursing services, thereby forming a positive nurse-patient interaction cycle. This study confirms the significant effect of narrative nursing in patients with hypertension.

It is also noteworthy that patients in the control group exhibited improvements in SDS/SAS scores and compliance after routine nursing. Several factors may explain this trend. First, although lacking narrative elements, conventional nursing included structured education, medication guidance, and emotional reassurance, which could contribute to modest positive changes. Second, the Hawthorne effect—where participants modify behavior due to the awareness of being studied—may have played a role. Finally, a degree of natural psychological adjustment during hospitalization may have occurred, as some patients gradually accept their condition and emotionally adapt to the healthcare environment. These effects underscore the importance of acknowledging the baseline impact of standard care while highlighting the added value of narrative nursing interventions.

This study is among the first to apply a comprehensive and structured narrative nursing framework—including externalization, deconstruction, rewriting, witnessing, and documentation—to patients with hypertension. The intervention protocol was standardized and fully completed by all participants, demonstrating high feasibility and acceptability in an inpatient setting. Outcome indicators covered psychological, behavioral, physiological, and satisfaction domains, providing a multi-dimensional evaluation of narrative nursing efficacy. Moreover, the approach offers a replicable and scalable model for managing chronic disease care in clinical practice. Future studies should consider conducting multi-center, prospective randomized controlled trials (RCTs) to enhance the robustness of evidence. Incorporating long-term follow-up data such as blood pressure variability, relapse rate, and quality of life measures will help determine sustained efficacy. It is also recommended to explore the integration of narrative nursing with digital health platforms (e.g., mobile apps, telehealth systems) to facilitate remote implementation. Additionally, applying this model to other chronic illness populations, such as diabetes and cardiovascular diseases, will help assess its generalizability and broader clinical utility.

### Limitations:.

First, it was a single-center, non-randomized retrospective study with a relatively small sample size, which may introduce selection bias and limit the strength of causal inferences. Although strict inclusion and exclusion criteria were applied to reduce heterogeneity, potential confounding factors—such as baseline psychological status, intrinsic motivation, and external social support—were not statistically controlled. Second, the short intervention duration and lack of long-term follow-up limited our ability to assess the sustained impact of narrative nursing on patient outcomes.

## CONCLUSIONS

The narrative nursing model in patients with hypertension can effectively alleviate depression and anxiety, significantly enhance treatment compliance, optimize blood pressure control and increase patients’ nursing satisfaction. This approach has good clinical application value and shows potential advantages in improving psychological adjustment and treatment compliance, but warrants further validation in larger, multi-center, and long-term studies.

### Recommendations:

Future studies should adopt multi-center, prospective randomized controlled designs with larger sample sizes, extended follow-up periods, and multivariable modeling to better evaluate the long-term effects of narrative nursing on quality of life and cardiovascular event risk in patients with hypertension. In addition, integrating narrative nursing with intelligent health management tools may offer opportunities for remote, dynamic, and personalized interventions, which warrants further exploration

### Authors’ contributions:

**LD:** Literature search, study design and manuscript writing.

**YS, YC, YW and HH:** Data collection, data analysis and interpretation. Critical review.

**LD:** Manuscript revision and validation and is responsible for the integrity of the study.

All authors have read and approved the final manuscript.
